# Entomological characterization of malaria in northern Colombia through vector and parasite species identification, and analyses of spatial distribution and infection rates

**DOI:** 10.1186/s12936-017-2076-5

**Published:** 2017-10-27

**Authors:** Camila González, Astrid Gisell Molina, Cielo León, Nicolás Salcedo, Silvia Rondón, Andrea Paz, Maria Claudia Atencia, Catalina Tovar, Mario Ortiz

**Affiliations:** 1 Centro de Investigaciones en Microbiología y Parasitología Tropical, CIMPAT, Departamento de Ciencias Biológicas, Universidad de los Andes, Cra. 1 No 18A-12, Bogotá, Colombia; 2 Facultad de Ciencias de la Salud, Universidad del Sinú, Cra 1w No 38-153, Montería, Colombia; 3 Grupo de Enfermedades Tropicales y Resistencia Bacteriana, Facultad de Ciencias de la Salud, Universidad del Sinú, Cra 1w No 38-153, Montería, Colombia

**Keywords:** *Anopheles*, DNA Barcode, Malaria, *Plasmodium falciparum*, *Plasmodium vivax*

## Abstract

**Background:**

Malaria remains a worldwide public health concern and, in Colombia, despite the efforts to stop malaria transmission, the incidence of cases has increased over the last few years. In this context, it is necessary to evaluate vector diversity, infection rates, and spatial distribution, to better understand disease transmission dynamics. This information may contribute to the planning and development of vector control strategies.

**Results:**

A total of 778 *Anopheles* mosquitoes were collected in fifteen localities of Córdoba from August 2015 to October 2016. Six species were identified and overall, *Anopheles albimanus* was the most widespread and abundant species (83%). Other species of the Nyssorhynchus subgenus were collected, including *Anopheles triannulatus* (13%), *Anopheles nuneztovari* (1%), *Anopheles argyritarsis* (< 1%) and two species belonging to the Anopheles subgenus: *Anopheles pseudopunctipennis* (3%) and *Anopheles neomaculipalpus* (< 1%). Four species were found naturally infected with two *Plasmodium* species: *Anopheles nuneztovari* was detected naturally infected with *Plasmodium falciparum* and *Anopheles pseudopunctipennis* with *Plasmodium vivax*, whereas *An. albimanus* and *An. triannulatus* were found infected with both parasite species and confirmed by nested PCR.

**Conclusions:**

In general, the obtained results were contrasting with previous studies in terms of the most abundant and widespread collected species, and regarding infection rates, which were higher than those previously reported. A positive relationship between mosquito local abundance at the locality level and human infection at the municipality level was found. Mosquito local abundance and the number of houses with mosquitoes in each village are factors explaining malaria human cases in these villages. The obtained results suggest that other factors related to the apparent variation in malaria eco-epidemiology in northern Colombia, must be identified, to provide health authorities with better decision tools aiming to design control and prevention strategies.

**Electronic supplementary material:**

The online version of this article (doi:10.1186/s12936-017-2076-5) contains supplementary material, which is available to authorized users.

## Background

Malaria transmission constitutes one of the greatest public health challenges on a global scale and the World Health Organization (WHO) has established an ambitious plan to work towards the control and elimination of the disease by 2030. The milestones set for 2020 include, among others, the reduction of case incidence and mortality rates by 40% [1]. In Latin America, between 2010 and 2015, there was a reduction of 31% in case incidence and of 37% of the mortality rate. Colombia ranked third after Venezuela (30%) and Brazil (24%) with 10% of the estimated malaria cases in the region.

Although efforts have been directed to meet these goals, in February 2017, the Pan American Health Organization issued an alert [2] due to a significant increase in malaria transmission in the Americas in 2016. Indeed, eight of the 21 endemic countries for disease transmission recorded an increase in the number of cases compared to 2015. Following the same pattern, Colombia reported a reduction of 72% in incidence and 87% in mortality between 2000 and 2014 [3], but in 2015 and 2016 the incidence of cases increased by 37% each year, reaching numbers similar to those found in 2006 [4].

In Colombia, *Plasmodium vivax* was historically reported as the most prevalent parasite species responsible for malaria cases in the country: for instance, in the 2010 outbreak, *P. vivax* represented 71% of the cases and *Plasmodium falciparum* 28% [5]. However, in 2016, the National System of Public Health Vigilance (SIVIGILA by its Spanish acronym) reported 83,356 cases of malaria in Colombia of which 47,497 (57%) were caused by *P. falciparum*, the parasite responsible for cerebral malaria, while 33,055 (40%) corresponded to *P. vivax* and 3.3% to mixed infection [4]. This increase in the number of cases caused by *P. falciparum* was also recorded in other countries, including Venezuela, Ecuador, Peru and Honduras, implying a higher risk of complicated malaria, and indicating failures in the access to treatment and in vector control [2].

According to the WHO [6], vector control is the main strategy to prevent and reduce malaria transmission, mostly through mosquito nets and indoor residual spraying. Entomological surveillance is the first step to make a proper assessment of vector species present in a transmission setting, as well as to establish infestation and infection rates. From this perspective, there is a need for conducting research related to malaria vectors in order to better understand disease transmission and to develop new control strategies [7], especially in the context of the epidemiological alert that is currently being faced [2].

Research on the eco-epidemiology of the disease in Colombia usually is made as a part of outbreak responses or short-term research programmes aiming to reduce the number of cases at a local scale [8–11]. Those studies highlight the entomological surveillance as a useful tool when planning strategies for malaria control.

In 2016, seven out of 32 Colombian departments (Chocó, Nariño, Antioquia, Amazonas, Guainía, Córdoba and Cauca), and the municipality of Buenaventura in Valle del Cauca department, recorded 92.8% of all malaria cases in the country. The department of Córdoba, recorded 1751 cases, and the municipality of Tierralta had the highest proportion of cases (56% of cases), followed by Puerto Libertador (24%), Montelibano (7.5%), Planeta Rica (1.7%) and Montería (1.6%). Córdoba is known for its cattle ranching practices and unplanned agriculture [12]; rice crops are present in this area [13] and *Anopheles pseudopunctipennis* has been found using them as breeding sites [14]. Previous entomological studies conducted in the department reported *Anopheles nuneztovari*, *Anopheles triannulatus*, *Anopheles darlingi*, *Anopheles albimanus* and *An. pseudopunctipennis*, among other species [15–17]. *An. nuneztovari* was the dominant species in those studies, and was considered as the primary malaria vector in the department [17].

In this context, this study was designed to investigate the entomological scenario of malaria in villages in Córdoba through the study of vector diversity, spatial distribution of vectors and parasites, and infection rates. The study design could be implemented as a tool to monitor vector-borne diseases occurring in domestic settings on a broad geographic scale in tropical regions, where baseline information on the situation of malaria needs to be assessed. In this way, this study can contribute valuable information to better design prevention and control strategies aiming to reduce the burden of the disease.

## Methods

### Entomological collection

**Fig. 1:**
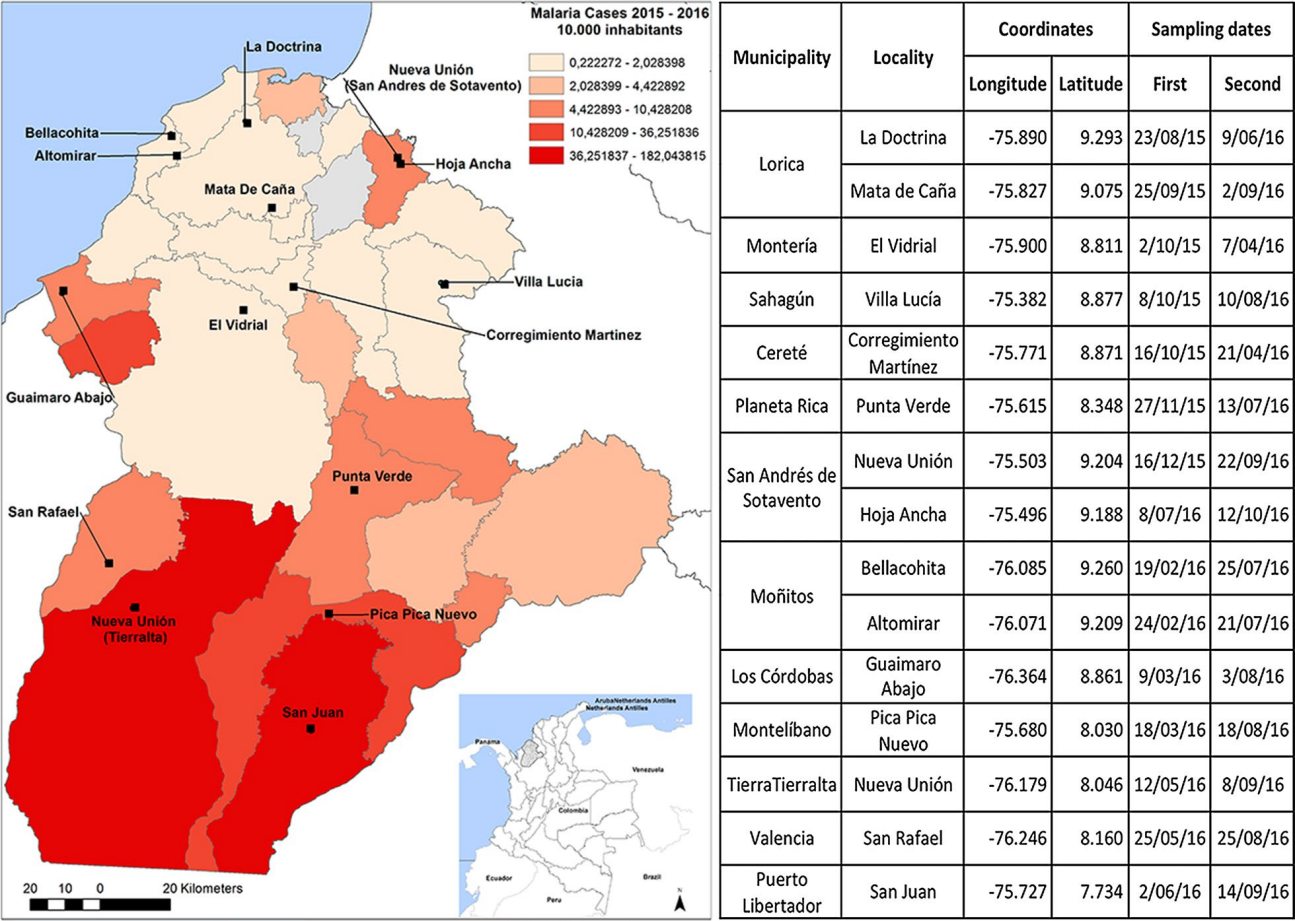
Study site showing sampled localities and collection dates. Black squares correspond to sampling locations in each locality. Table shows the location and dates for each sampled locality. Coordinates correspond to the 12th house of each locality and sampling dates to the first night of sampling out of three consecutive nights

Fifteen villages within 13 municipalities of the Department of Córdoba, Colombia (09°26′16″ N, 76°30′01″ W), were selected for sampling based on different criteria: their epidemiological importance in terms of vector-borne diseases, accessibility, and security (Fig. 1). Entomological collections within each town were performed in 24 houses, randomly selected to establish potential malaria vector species present in the department. The selection of households was performed using a 25 m × 25 m grid created using ArcMap 10.1 [18] on top of a satellite image of the locality from Google Earth [19] covering all the area with houses. Inside the grid, 24 cells were randomly selected for sampling. For the seven localities with no available satellite images (Villa Lucia-Sahagún, San Juan—Puerto Libertador, Hoja Ancha—San Andrés De Sotavento, Punta Verde—Planeta Rica, Altomirar—Moñitos, Guaimaro abajo—Los Córdobas and Pica Pica—Montelibano) all houses were georeferenced in an exploratory field trip and 24 were randomly selected using R [20].

Houses were numbered from one to 24 and eight houses were sampled each night during three consecutive nights using two CDC light traps; one was located indoors, and the other one, outdoors. In odd numbered houses (e.g. house 1, 3 and 5), a BG Sentinel trap baited with BG-Lure [21] was placed inside. CDC traps were set at 6:00 p.m. and left active for 12 h, and BG Sentinel traps were active for 24 h. The following morning, traps were revised and mosquitoes were sorted for further taxonomic and molecular analyses. During the day, households were examined for breeding sites, and diurnal adult collections were performed using backpack aspirators [22]. Each town was sampled twice between August 2015 and September 2016 (Fig. 1).

All male mosquitoes were preserved dry in tubes with silica gel for their accurate taxonomic identification. All female mosquitoes were stored in 70% ethanol to perform molecular analyses for parasite detection. Species identification was performed based mainly on morphological characters of males, although, females, preserved in liquid, were also examined for general morphological characteristics using the keys of González and Carrejo [23], Lane [24], and Forattini [25]. Due to the loss of important taxonomic characters, for 65 specimens we extracted DNA from legs (or abdomen tissue in individuals lacking appendages) and performed species identification using DNA barcoding trough amplification of the 658 bp region from the COI gene. The PCR mix consisted of 1 µL of total DNA, 12.5 µL of 2 × GoTaq Green Master Mix (Promega), and 10 µM of the primers LCO1490 and HCO2198 [26]. The cycling conditions were: 1 min at 94 °C; followed by 40 cycles of 40 s at 94 °C, 40 s at 52 °C, 1 min at 72 °C; and a final extension of 5 min at 72 °C. All PCR products were sequenced at the DNA sequencing laboratory UNIANDES using the ABI-3500 Genetic Analyzer (Life Technologies).

Analysis by means of ANOVAS were made to evaluate differences on *Anopheles* collections between indoor and outdoor traps, and a post hoc test (Tukey test) was performed to assess the effect of locality on mosquito’s abundances.

### Parasite detection and species identification

To detect the presence of parasites, DNA was extracted from pools of up to ten female *Anopheles* mosquitoes from the same species, using ZR Tissue & Insect DNA Miniprep kit (Zymo) following the manufacturer’s manual. *Plasmodium* sp. detection was performed through nested PCR following previously published methods [27] using GoTaq Green Master Mix (Promega). All PCR products from second reactions were visualized on an agarose gel, targeting a 205 bp fragment for *P. falciparum* and a 120 bp fragment for *P. vivax*, positive samples were sequenced to confirm species identity. Sequences were cleaned and aligned using *CLC Genomics Workbench 3.6.5 Software* and compared with publicly available sequences using BLAST. To confirm the identity of the *Anopheles* species present in all the positive pools, 1 µL of the pooled DNA extract was used and the barcode technique was applied as explained before.

### Eco-epidemiological and spatial analyses

For each sampled town, the minimum infection rate (MIR) for each *Anopheles* species and for all species together was computed, based on the assumption that each positive pool should contain at least one infected mosquito [28]. Using the number of positive houses (i.e. household with at least one mosquito pool infected with *Plasmodium*) a ratio was calculated for each town.

To evaluate if there was a threshold of mosquito abundance that could sustain parasite presence, Pearson correlations between mosquito abundances and the number of positive houses were assessed (after testing for normality of all variables with a Shapiro–Wilk test). Furthermore, multiple regression models were fitted to identify the variables that influence the number of infected inhabitants.

Regarding spatial analyses, to evaluate if the distribution of collected and infected mosquitoes was random in space, Moran’s Index calculations were performed in ArcGIS 10.2. For this, a Global Moran’s I where the null hypothesis is that of random distribution in space was computed for each of two feature classes, the first describing the number of infected pools per municipality and the second the number of mosquitoes per municipality. The relationship between infection and environment was also evaluated. For this, sampled localities, positive localities for *Anopheles* and localities with mosquitoes infected with *Plasmodium* species were intersected with land cover information [29].

To assess temporal variation in mosquito collections, seasonality was evaluated by associating the collection dates to precipitation patterns in the Department described by Guzmán et al. [30]; precipitation is the key element defining seasonality in the tropics since temperature is relatively stable.

## Results

### Entomological collection

A total of 778 *Anopheles* were collected, 42 males and 736 females, with the genus being present in all the sampled localities. The CDC light traps were the most efficient: those placed outdoors collected 406 individuals while indoor traps collected 355. Aspirator collection added 13 individuals while we only caught four *Anopheles* in BG Sentinel traps. There were no significant differences between the average number of *Anopheles* collected indoors or outdoors either for total counts (*p* = 0.519) or individual species *An. albimanus* (*p* = 0.634), *An. triannulatus* (*p* = 0.663). A significant effect of locality on the number of mosquitoes was detected for total counts (*p* = 0.0025); a post hoc Tukey test revealed this effect was explained by the difference between San Juan and Hoja Ancha and the difference between San Juan and San Rafael. The interaction of mosquito house collection point (indoors or outdoors) and locality was non-significant (*p* = 0.866).

**Fig. 2:**
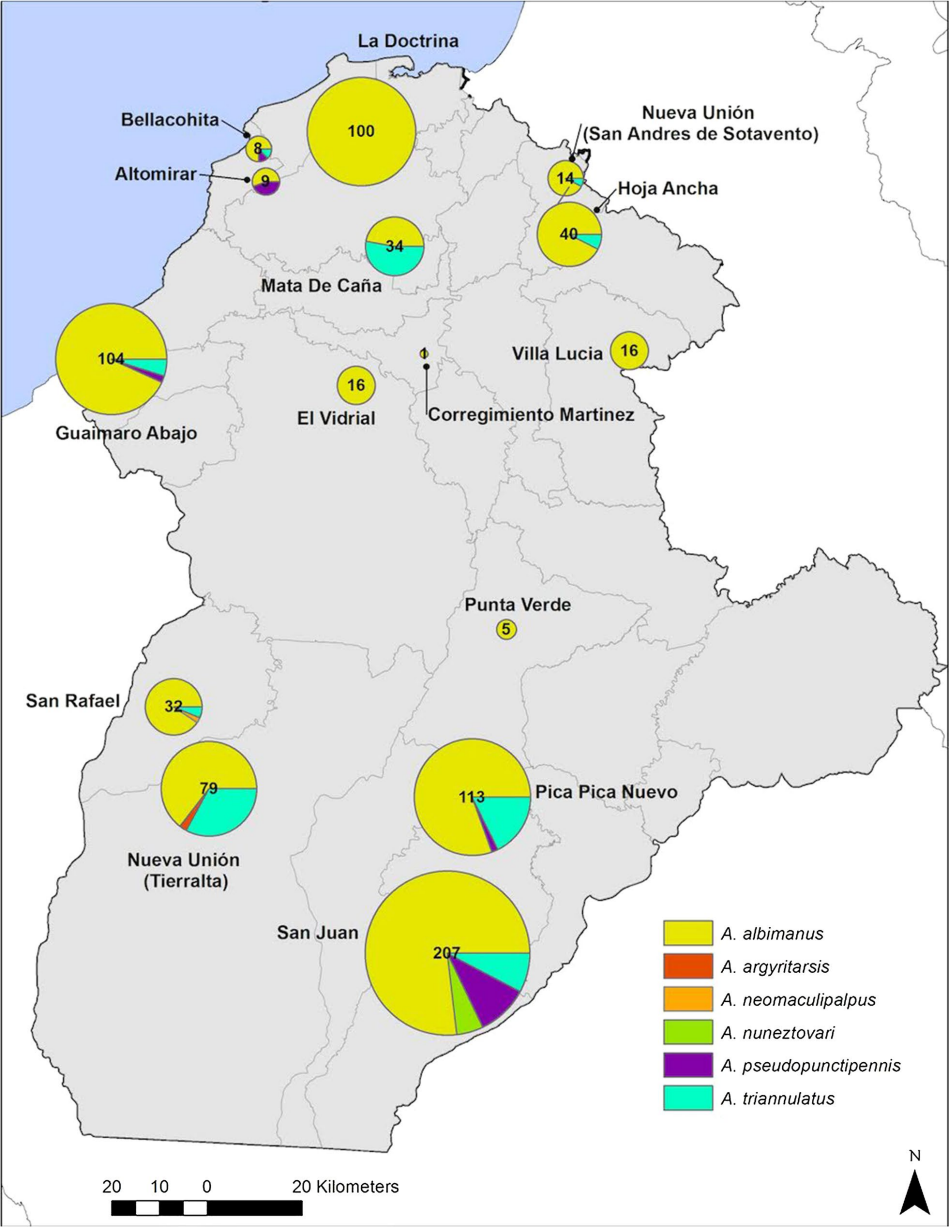
Proportion of local abundances for the six collected *Anopheles* species in each locality over the total number of *Anopheles* collected

Five localities accounted for 77.5% (n = 603) of the collected *Anopheles* females: San Juan (27%), Pica Pica Nuevo (14.5%), Guaimaro Abajo (13%), La Doctrina (13%) and Nueva Unión—Tierra Alta (10%) (Fig. 2).

Six *Anopheles* species belonging to two subgenera: *Nyssorhynchus* (four species) and *Anopheles* (two species) were identified. *Anopheles albimanus* was found in every vector-positive locality, and was the predominant species, corresponding to 83% of the total identified specimens. *Anopheles triannulatus* accounted for 12%, and four other species accounted for 4% of the identified vector species: *A. pseudopunctipennis* (4%), *An. nuneztovari* (1.3%), *Anopheles neomaculipalpus* (0.26%) and *Anopheles argyritarsis* (0.13%) (Fig. 2, Additional file 1: Table S1).

Specimens were identified as: 110 *An. albimanus*, 32 *An. triannulatus*, 10 *An. pseudopunctipennis*, 9 *An. nuneztovari* and 2 *An. neomaculipalpus.* Barcode sequences were obtained for 65 specimens (for identification) and 37 pools (for species confirmation); specimens were identified by comparing obtained sequences to available sequences on GenBank [31]. DNA barcode analyses allowed for the identification of nine individuals that were not identified using morphology; six individuals corresponded to *An. albimanus* and three to *An. triannulatus*. A total of 35 individuals were found to have been misidentified using morphology (an error of 10.7% in morphological identification). Twenty-six specimens previously identified as *Anopheles benarochi* were rectified as *An. albimanus* (14) and *An. triannulatus* (12). Also, nine *An. albimanus* were rectified to *An. nuneztovari* (Table 1).

**Table 1:** Reference sequences used for comparison of DNA barcodes generated in this study, including species name, GenBank number, % identity threshold used for identifications and sources of the reference sequences

Species	GenBank sequence	% identity (%)	Origin
*A. pseudopunctipennis*	KC354819.1	98	[32]
*A. neomaculipalpus*	JX205125.1	98	[33]
*A. albimanus*	KC354823.1	99	[32]
*A. triannulatus*	JX205112.1	98	[33]
*A. nuneztovari*	KU865556.1	99	[34]

### Parasite detection and species identification

**Fig. 3:**
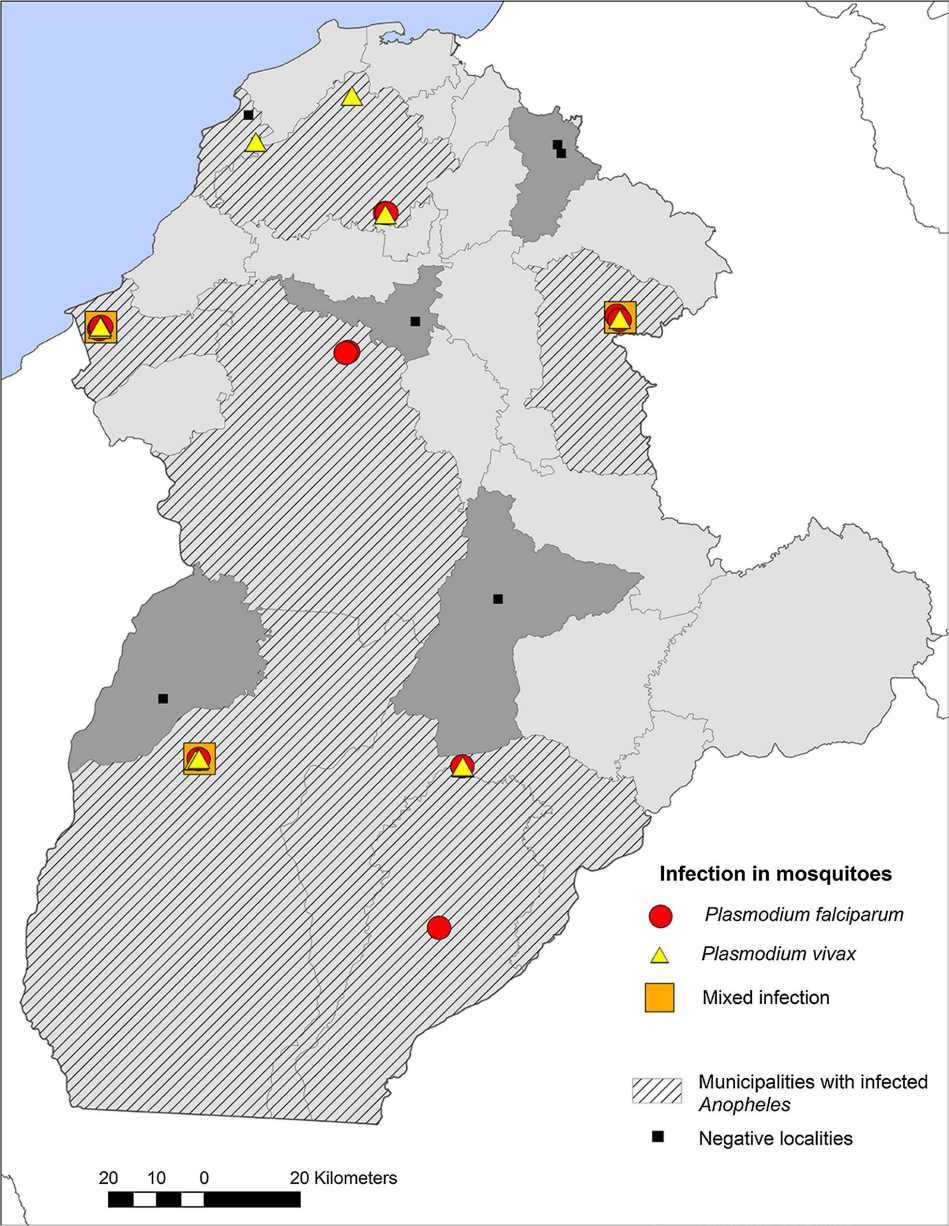
Distribution of infected mosquitoes and *Plasmodium* species found in each locality; positive municipalities are represented by simple hatch and negative localities by the black square symbol

Of the fifteen sampled localities, nine were positive for two species of *Plasmodium*, *P. vivax* and *P. falciparum.* Two localities were positive for only *P. vivax* (Altomirar and La Doctrina), and two localities only for *P. falciparum* (San Juan and El Vidrial), the remaining five localities were positive for both parasites, and in three of them mixed infections in *An. albimanus*, pooled from the same house, were detected (Fig. 3).

Mosquitoes collected in 38 households were found to be positive for parasites, 13 exclusively for *P. falciparum* (34.2%) with 15 positive pools, 23 for *P. vivax* (57.8%) with 19 positive pools, and three for mixed infection with three positive pools (7.8%). Five DNA samples produced a high number of non-specific PCR products when screened for *P. falciparum*, in those cases no sequence was obtained and they were therefore designated as ‘failed reactions’. All positive PCR results were confirmed by sequencing [35] (Fig. 3).

Four *Anopheles* species were found infected with *Plasmodium*; the positive pools were more commonly *An. albimanus* (31 positive pools), followed by *An. triannulatus* (Four positive pools). One pool of *An. nuneztovari* was found infected with *P. falciparum* and one pool of *An. pseudopunctipennis* with *P. vivax* (Fig. 3) (Additional file 2: Table S2).

### Eco-epidemiological and spatial analyses

According to our analyses, no positive correlation was found between the local abundances of *Anopheles* and the number of positive houses (ρ = 0.089; p value 0.205). *Anopheles* mosquitoes were present in all sampled towns with one species, *An. albimanus*, dominating across the whole department and being present in every sampled locality (Fig. 2). The geographic distribution of *Anopheles* mosquitoes in the municipalities of Córdoba was random in space (Morans I = 0.0054, z-score = 0.54, *p* value = 0.59). *Anopheles triannulatus* was found in nine localities of eight municipalities and, as was also the case for *An. pseudopunctipennis*, which was present in four municipalities, was spatially scattered. *Anopheles neomaculipalpus* was only present in two of the sampled municipalities (Los Córdobas and Valencia), while *Anopheles argytarsis* was found only in the south of Córdoba in the municipality of Tierra Alta. Interestingly, *An. nuneztovari*, the most important species in previous studies [36, 37] was only present in Puerto Libertador. Regarding breeding sites, no *Anopheles* larvae were found in water sources close to sampled houses.

The number of females per locality was highly variable ranging from one to 159 individuals and no site had all six species coexisting. The most diverse communities had different combinations of four mosquito species maximum. The town of San Juan in the municipality of Puerto Libertador had the highest abundance of mosquitoes (n = 154), and a fairly diverse community of four different species: *An. albimanus*, *An. nuneztovari*, *An. pseudopunctipennis*, and *An. triannulatus*.

Interestingly, mosquito abundances were related to sampling locality (p value two-way anova = 0.0025). This effect however, was limited to the localities of San Juan in the municipality of Puerto Libertador and San Rafael in the municipality of Valencia, with the first town having 3.16 times (S.E = 0.85) more mosquitoes per night per house than the latter.

Sampling was performed in areas classified in either of five categories according to the CORINE classification: urban areas, transitory cultures, grasses, agricultural areas, and continental water areas. Mosquito presence was registered in all sampling areas (background). Distribution of infected mosquitoes was geographically scattered in Córdoba (Morans I = − 0.04, z-score 0.0013, *p* value = 0.99) although presence of infected mosquitoes was disproportionately higher in areas covered by grasses (mainly used for cattle) in relation to the overall grass coverage in the sampling areas. For other land cover categories, the presence of infected mosquitoes was either proportional to the sampling background or lower.

In terms of seasonality, the number of collections varied during the seasons and more species were collected during the rainy season (six) than during the dry season (three), however, only five localities could be sampled in both seasons due to logistic difficulties. Based on these localities, the average number of mosquitoes collected in each season for each species was higher in the rainy season for *An. albimanus* (8.6 in rainy season, 8.2 in dry season), *An. triannulatus* (6.2 in rainy season, 1 in dry season) and *An. pseudopunctipennis* (1.6 in rainy season, 1 in the dry season). The remaining species *An. argyritarsis*, *An. neomaculipalpus* and *An. nuneztovari*, were only collected in the rainy season.

## Discussion

Overall, the obtained results are contrasting with previous entomological studies performed in Córdoba between the years 2008 and 2014, which reported *An. nuneztovari* as the most abundant species; in this study it accounted only for 1.3% of collected specimens and was only found in one locality and only in the rainy season [15–17]. The most abundant and widespread species was *An. albimanus*, found in every sampled locality and both seasons. This species is of epidemiological importance since it is considered as a primary vector in the low coastal regions of the Caribbean and Pacific coasts in Colombia [17], and our results contribute the first records of this species in two localities, Los Cordobas and Puerto Libertador. The second most abundant species, *An. triannulatus*, was originally described as zoophilic, and is widely distributed in Colombia [23]. Although it has been incriminated as local or regional vector in neighboring countries, its incrimination in Colombia has not been determined yet, but Gutierrez et al. suggested that this species could be acting as secondary malaria vector due to the high infection rates it exhibits [30, 36, 38, 39]. Rosero et al. [39] reported this species in northern Colombia, collected in Córdoba only in the rainy season of 2010, and occurring, as found in this study, in sympatry with primary malaria vectors such as *An. albimanus*, *An. nuneztovari* and *An. darlingi* and they detected *An. triannulatus* infected both with *P. vivax* and *P. falciparum*. An effect of rain seasonality on species composition was found in the present study; since community composition and abundances are related to the sampling season, generating a complete survey requires multi-season sampling. Furthermore, caution must be taken when comparing studies performed at different seasons. For example, the study of Rosero et al. found that in Córdoba, *An. nuneztovari* was the dominant species during the rainy season in August and November 2009 and June 2010 whereas the dominant species in the dry season of February 2010 was *An. pseudopunctipennis*.

*Anopheles pseudopunctipennis* was found infected with *P. vivax;* this species was previously collected in the south of Córdoba but always in lower abundances compared to those found in this study [16, 17]; this species is here reported for the first time in the northern part of the department, in the municipality of Moñitos. This scenario could be reflecting the effect of the sampling methods, using exclusively traps for insect collection, but might be providing valuable information that should be deepened regarding changes in the ecology of disease transmission related to the current changes in malaria epidemiology recorded in the Americas [2].

For instance, the proportion of *P. falciparum* detected when compared to previous studies seems to be increasing, although it is still lower than *P. vivax* (Table 2). Both species were detected sympatrically, occurring in the same localities, and even three pools of *An. albimanus* were detected with mixed infection. However, mixed infections in mosquitoes seem not to be critical from the epidemiological perspective, since mixed infections in humans seem to be the result of multiple inoculations (Table 2) [40].

**Table 2:** Results of infection by locality showing the minimum infection rate (MIR), calculated as a ratio, considering one positive mosquito per pool, proportion of positive houses, and parasite species detected

Village	Total *Anopheles* screened	Infected pools	MIR (ratio)	Proportion of positive houses			
				Total over 24 sampled houses	Positive for *P. vivax*	Positive for *P. falciparum*	Positive for mixed infection
Alto Mirar	9	1	0.11	0.04	0.04	0	0
El Vidrial	12	2	0.17	0.08	0	0.08	0
Guaimaro abajo	100	12	0.12	0.63	0.42	0.17	0.04
La Doctrina	90	1	0.01	0.04	0.04	0	0
Mata de Caña	32	3	0.09	0.13	0.04	0.08	0
Nueva Unión	76	8	0.11	0.33	0.25	0.04	0.04
Pica pica nuevo	108	5	0.05	0.13	0.08	0.04	0
San Juan	198	1	0.01	0.04	0	0.04	0
Villa Lucia	15	4	0.27	0.17	0.05	0.1	0.04

Malaria transmission in the Department during the time of our study, as recorded by the SIVIGILA in 2015–2016, included 3195 cases of malaria, with Tierralta contributing 58%, Puerto Libertador 20% and Montelibano 6%. One case of mixed infection from Puerto Libertador was detected, while *P. falciparum* caused 11 cases (in Tierralta, Valencia and Planeta Rica), and *P. vivax* caused 48 cases (in Tierralta, Puerto Libertador, Canalete and Montelibano). Transmission areas where human cases were reported were also areas where collected *Anopheles* were infected with parasites. In Puerto Libertador, *P. falciparum* was detected in one pool of *An. nuneztovari*, while in Montelibano and Tierralta *An. albimanus* was found infected with *P. falciparum* and *P. vivax*. However, the locality of Los Cordobas which in this study exhibited the highest number of infected pools with both parasite species, did not record human cases. This situation could be indicating that population is acting as an asymptomatic reservoir, so asymptomatic infections must be taken into account, in the same way that symptomatic infections, involve circulating gametocytes which can infect mosquitoes even at low densities, contributing to parasite transmission [41].

In general, MIRs found in this study were much higher than those found, for example, by Ahumada et al. [8]; this could be the result of our preservation method (absolute ethanol), which has shown to be more efficient than silica gel, as high concentrations of ethanol can denature proteins which degrade DNA [42]. In addition, the method standardized by Rath et al. [43] was performed for *Plasmodium* spp. identification by pools of up to 10 individuals, which can increase the detection capacity compared to individual extraction. Also, the methodology by Snounou et al. [27] was followed, and sequencing was carried out in order to confirm the amplified products; in spite of the high numbers, the detected infection rates in the present study are reliable. An important comment on the detected infection rates, is that whole mosquitoes were used to perform parasite detection, and for the incrimination of malaria vectors it is advisable to use only head and thorax, to demonstrate the species ability to be infectious [43, 44]. In this way, it cannot be assumed that infected mosquitoes could subsequently transmit malaria parasites and act as vectors.

In the present study, six *Anopheles* species were collected in Córdoba, all of them occurring in human settlements in urban localities, and were equally collected inside and outside houses. This number of species and the number of collected specimens is small when taking into account that 47 species of *Anopheles* are recorded in the country [43], 20 species are known to occur in the Department [23] from the 47 species of *Anopheles* recorded in the country [45], and that previous studies have collected thousands of *Anopheles* using human-landing catches [11, 36]. Regarding collection numbers and species composition, this could be affected by the use of light traps or traps artificially baited. Human-landing catches has been the most commonly used method in malaria studies [15, 16, 36, 46, 47], however, it have been controversial due to ethical considerations, since it threatens the subject acting as bait [48]. For the purpose of large-scale sampling and comparative studies, the use of traps is advised. Qiu et al. [49] performed a study using Mosquito Magnet® traps with the addition of CO_2_ to a blend of ammonia + l-lactic acid + 3-methyl-butanoic acid as synthetic odors which considerably increased the capture of anthropophilic Anophelines as well as other mosquito species; additionally, Mosquito Magnet® trap has an advantage over the CDC light trap and the BG-Sentinel® as it can function for weeks without batteries or propane replacement. However, an optimum performance trap for *Anopheles* collection that does not compromise the quality of the samples is still required. Based on previous studies [50, 51], we expected the BG sentinel traps to be the more efficient, however, CDC light trap, performed better, possibly due to the low efficacy of the attractants used in the BG sentinel traps [49].

Regarding species identification, all the samples subjected to barcode analyses exhibited 98–99% sequence identity to published sequences, 98% for *An. triannulatus* and *An. neomaculipalpus* [33], 98% for *An. pseudopunctipennis* [52] and 99% for *An. albimanus*, and 99% for *An. nuneztovari* [34]. Concordance between molecular and morphological identification can be used as an indication of reliability of the overall results, and it can help in species identification of species complex, such as *An. triannulatus* and *An. nuneztovari.*

The possibility of using DNA barcodes to confirm species identification is strongly recommended [53, 54]. Although these analyses can be expensive to perform in developing countries, they allow optimizing through molecular biology, the information gathered from valuable field samples such as species identification, infection and blood source analyses, critical steps to perform vector species incrimination. From our experience, it is also important to establish adequate preservation procedures for the collected samples. Insect samples should be transported frozen [55] as an alternative to ethanol since insects kept in ethanol solution suffer the loss of important characters used in their morphological identification, such as legs, scales and setae [56].

Entomological collections were made in villages, surrounded by agricultural matrices and disturbed ecosystems, so deforestation and land use changes can influence vector species composition and diversity. It is known that agriculture has an impact on the establishment of transmission cycles of vector-borne diseases, particularly affecting the availability of breeding sites; sampling localities coincided mostly with grasses and agricultural settings (both permanent and transitory) with positive sites being disproportionately associated with grasses [57]. With the obtained results, looking for breeding sites could provide an important contribution to be able to relate species occurrences and land coverage, especially in changing scenarios.

Lastly, no correlation was found between rain seasonality and species abundances or composition. The adult abundance of *An. albimanus* was, with few exceptions, directly related to high precipitation [58] and nearly disappeared during the dry season [59].

## Conclusions

The most abundant and widespread species found in our study was *An. albimanus*, a result contrasting with previous studies; it is important to establish whether this difference is due to bias in the type of traps used, or if indeed there is a variation in species composition and distribution possibly due to climate changes. Further studies should also assess the role of *An. triannulatus* in malaria transmission since its role has not been established yet but has been found infected.

The infection rates found in mosquitoes using molecular analysis, and subsequent sequencing are higher than those reported in previous studies. The use of DNA barcoding as a tool for support taxonomic identification is advised, when inadequate storage of mosquitoes impedes morphological identification.

## Additional files

**Additional file 1.** *Anopheles* database including capture sites, geographic location and gender.

**Additional file 2.** Localities where positive pools were detected showing the total number of *Anopheles* screened, positive pools, parasite species, and minimum infection rate.
