# Supplementary material for: Entomological characterization of malaria in northern Colombia through vector and parasite species identification, and analyses of spatial distribution and infection rates
Source: Malar J. 2017 Oct 27;16:431. doi: 10.1186/s12936-017-2076-5 (PMC5658941; doi:10.1186/s12936-017-2076-5)
Supplement: Supplementary file 1 — Additional file 1. Anopheles database including capture sites, geographic location and gender. [file 12936_2017_2076_MOESM1_ESM.docx]

| **Village** | **Total screened** | **Anopheles species** | **Positive pools** | **Parasite species** | **Minimum infection rate** |
| --- | --- | --- | --- | --- | --- |
| Guaimaro abajo | 5 | *Anopheles triannulatus* | 1 pool (1 individual) | *Plasmodium vivax* | 20 |
|  | 100 | *Anopheles albimanus* | 1 pool (5 individuals) | *Plasmodium vivax* | 12 |
|  |  |  | 2 pools (3 individual) |  |  |
|  |  |  | 3 pools (7 individuals) |  |  |
|  |  |  | 2 pools (1 individual) | *Plasmodium falciparum* |  |
|  |  |  | 1 pool (4 individuals) |  |  |
|  |  |  | 1 pool (6 individuals) |  |  |
|  |  |  | 1 pool (3 individuals) | *Plasmodium falciparum/vivax* |  |
| La Doctrina | 90 | *Anopheles albimanus* | 1 pool (11 individuals) | *Plasmodium vivax* | 1.1 |
| Mata de Caña | 18 | *Anopheles triannulatus* | 1 pool (1 individual) | *Plasmodium vivax* | 5.6 |
|  | 14 | *Anopheles albimanus* | 1 pool (1 individual) | *Plasmodium falciparum* | 14.3 |
|  |  |  | 1 pool (1 individual) |  |  |
| Alto Mirar | 9 | *Anopheles albimanus* | 1 pool (1 individual) | *Plasmodium vivax* | 11.1 |
| Pica pica nuevo | 87 | *Anopheles albimanus* | 1 pool (10 individuals) | *Plasmodium falciparum* | 3.4 |
|  |  |  | 1 pool (11 individuals) |  |  |
|  |  |  | 1 pool (2 individuals) | *Plasmodium vivax* |  |
|  | 20 | *Anopheles triannulatus* | 1 pool (1 individual) | *Plasmodium falciparum* | 5 |
|  | 1 | *Anopheles pseudopunctipennis* | 1 pool (1 individual) | *Plasmodium vivax* | 100 |
| El Vidrial | 12 | *Anopheles albimanus* | 1 pool (1 individual) | *Plasmodium falciparum* | 16.7 |
|  |  |  | 1 pool (2 individuals) |  |  |
| San Juan | 198 | *Anopheles nuñeztovari* | 1 pool (9 individuals) | *Plasmodium falciparum* | 0.5 |
| Villa Lucia | 15 | *Anopheles albimanus* | 1 pool (1 individual) | *Plasmodium falciparum* | 26.7 |
|  |  |  | 1 pool (4 individuals) |  |  |
|  |  |  | 1 pool (1 individual) | *Plasmodium falciparum/vivax* |  |
|  |  |  | 1 pool (1 individual) | *Plasmodium vivax* |  |
| Nueva Unión | 26 | *Anopheles triannulatus* | 1 pool (1 individual) | *Plasmodium vivax* | 3.8 |
|  | 50 | *Anopheles albimanus* | 1 pool (1 individual) | *Plasmodium falciparum* | 14 |
|  |  |  | 1 pool (2 individuals) | *Plasmodium falciparum/vivax* |  |
|  |  |  | 1 pool (4 individuals) | *Plasmodium vivax* |  |
|  |  |  | 1 pool (5 individuals) |  |  |
|  |  |  | 1 pool (1 individual) |  |  |
|  |  |  | 2 pool (3 individuals) |  |  |
